# Comparison of PrASE and Pyrosequencing for SNP Genotyping

**DOI:** 10.1186/1471-2164-7-291

**Published:** 2006-11-16

**Authors:** Max Käller, Emilie Hultin, Kristina Holmberg, Marie-Louise Persson, Jacob Odeberg, Joakim Lundeberg, Afshin Ahmadian

**Affiliations:** 1 Department of Biotechnology, Royal Institute of Technology (KTH), AlbaNova University Center, Roslagstullsbacken 21, SE – 106 91 Stockholm, Sweden; 2 Clinical Chemistry Laboratory, Blekinge Hospital, Karlskrona, Sweden; 3 Department of Medicine, Atherosclerosis Research Unit, King Gustaf V Research Institute, Karolinska Institutet, Karolinska University Hospital, Stockholm, Sweden; 4 Stanford Genome Technology Center, Stanford University, Palo Alto, CA 94304, USA

## Abstract

**Background:**

There is an imperative need for SNP genotyping technologies that are cost-effective per sample with retained high accuracy, throughput and flexibility. We have developed a microarray-based technique and compared it to Pyrosequencing. In the protease-mediated allele-specific extension (PrASE), the protease constrains the elongation reaction and thus prevents incorrect nucleotide incorporation to mismatched 3'-termini primers.

**Results:**

The assay is automated for 48 genotyping reactions in parallel followed by a tag-microarray detection system. A script automatically visualizes the results in cluster diagrams and assigns the genotypes. Ten polymorphic positions suggested as prothrombotic genetic variations were analyzed with Pyrosequencing and PrASE technologies in 442 samples and 99.8 % concordance was achieved. In addition to accuracy, the robustness and reproducibility of the technique has been investigated.

**Conclusion:**

The results of this study strongly indicate that the PrASE technology can offer significant improvements in terms of accuracy and robustness and thereof increased number of typeable SNPs.

## Background

It is now a common belief that single nucleotide variations in the human genome are responsible for influencing traits such as differences in drug metabolism and disease risk. These variations are referred to as single nucleotide polymorphisms (SNPs) and several large-scale technologies have recently been developed for scoring of thousands of SNPs and approaching whole-genome genotyping [1-5].

However, for smaller scale projects where potential genes are already known, technologies for genotyping of many samples instead of SNPs and in addition retain high accuracy and throughput, are more attractive compared to assays that are cost effective per SNP. A flexible choice of SNPs is also important instead of a pre-defined set of SNPs. There are several technologies already used in academic contexts but the earliest paralleled assays relied upon hybridization of short allele-specific probes to the target DNA [6,7]. However, improvements in microarray-based technologies in terms of accuracy have been achieved by enzymatic means [8-10]. One of these technologies involves allele-specific extension (ASE) which utilizes the ability of DNA polymerase to distinguish matched and mismatched 3'-termini of primers. However, a number of reports have shown that some mismatched 3'-ends can be elongated, giving false positive signals [11-13]. Nevertheless, as previously described, by exploiting the fact that the mismatched primers have slower reaction kinetics, the problems associated with ASE can be circumvented by including a protease (Proteinase K) that degrades the polymerase [14]. In the protease-mediated allele-specific extension (PrASE), the protease constrains the elongation reaction and thus prevents incorrect nucleotide incorporation to mismatched 3'-termini primers.

In this work, an automated PrASE assay with a tag-microarray detection system has been used and a comprehensive comparison of genotyping results with Pyrosequencing [15,16] has been done. Ten polymorphic positions previously studied with Pyrosequencing [17] for their suggested association as prothrombotic genetic variations [18-20] were used (Table 1, amplicon GC contents of 38 to 69%). These were analyzed on genomic DNA from unrelated DNA samples of Caucasian/Scandinavian origin from a cohort of patients presenting with symptoms of acute chest pain [17]. A total of 4420 genotypes were scored by each method and accordingly this study offers a thorough characterization of a microarray-based technique in terms of accuracy, robustness and variability.

## Results and discussion

**Figure 1:**
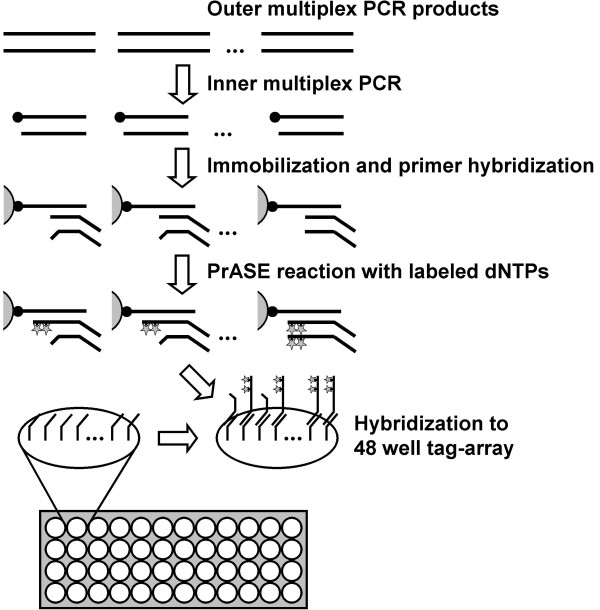
**SNP typing by PrASE**. A nested multiplex PCR is performed to amplify all SNP loci in a single reaction. The biotin-labeled inner PCR products are captured by streptavidin-coated magnetic beads facilitating automated reaction clean-ups between all assay steps. Strand-specific alkali elution is then performed before hybridization of allele-specific extension primers which contain unique tag sequences for later microarray detection. The multiplex PrASE reaction is performed with Cy5-labeled dNTPs to facilitate fluorescence detection. The products of the reaction are released with alkali, neutralized, and hybridized to a universal tag microarray containing 48 identical wells before detection.

**Figure 2:**
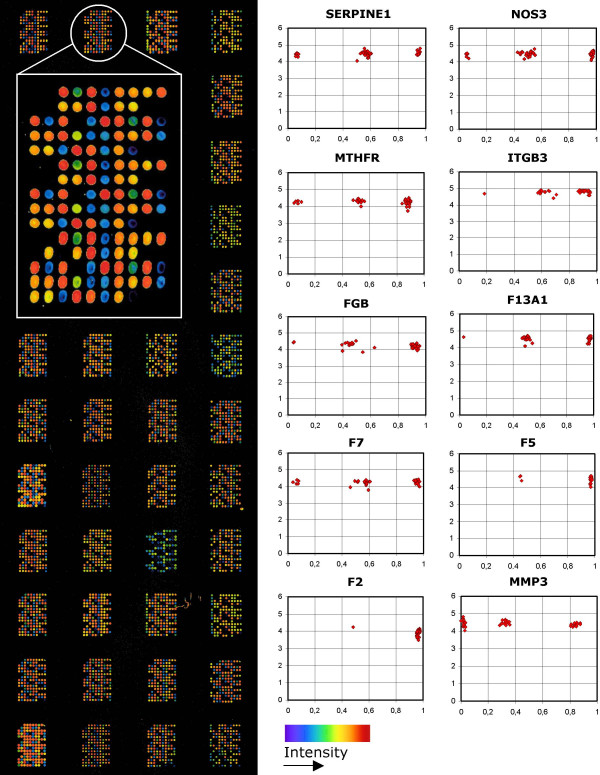
**An array-of-microarrays and the corresponding clusters**. 48 subarrays are spotted to each glass each having the entire set of 40 anti-tags spotted in triplicates (left panel insert) thus constituting an array-of-arrays (left panel). The colors are artificially added according to signal intensity by the software. The two signals for each SNP are plotted together as cluster diagrams where the three clusters represent each of the three possible genotypes (right panel). Allelic fractions (AFs, x-axes) are calculated from the signal intensities as spot 1/(spot 1 + spot 2) and logarithm of the total signal is used for the y-axes. To investigate the variability between tag sequences, each allele-specific extension primer was designed with two alternative tag sequences. All combinations for each SNP gave similar clusters to the presented.

The PrASE assay employed for parallel genotyping of SNPs is outlined in Figure 1 with some minor modifications to the original protocol [14], see Methods for details. Extracted genomic DNA is amplified in a nested PCR to achieve high specificity as well as to avoid extensive optimization of the multiplex amplification. Amplicons of minimal and similar sizes (48–58 bp) were chosen for the inner PCR to minimize the amount of PCR optimizations. The amplification products are immobilized on magnetic beads via biotin-streptavidin binding. By using a solid phase reaction, full automation of 48 genotyping reactions in parallel could be facilitated with a magnet-equipped pipetting robot. The washes of the 12 robot tips were now optimized to keep contaminations between sample rows undetectable (see description of results below). The genotyping protocol takes approximately 2.5 hands-off hours and 30 minutes of hands-on. This was followed by a one hour microarray detection of the 48 samples on one standard slide. In brief, after PCR product immobilization and clean-ups, the allele-specific extension primers are hybridized to ssDNA and the multiplex PrASE reaction is carried out by use of Cy5 labeled dNTPs, allowing fluorescence detection. The products of the PrASE reaction are then hybridized to a tag-microarray via unique tag-sequences included in the extension primers. The generic signature tag-arrays allow the genotyping results for the SNPs to be separated into different spots. A custom made silicone rubber mask was used to divide each slide into 48 identical wells (an array of microarrays), facilitating analysis of 48 samples in parallel. The fluorescence signals for each pair of allele-specific primers were counted with an image analysis software. A script automatically visualizes the results as cluster diagrams for each SNP and genotypes the samples by calculating allelic fractions (AFs). AFs are set to be the intensity from allele 1 (i.e. spot 1) divided by the sum of intensities from both alleles. The AFs are then plotted for each SNP against the logarithm of the sum of both signals. An example of a raw data image of an entire slide and the corresponding cluster diagrams for the 48 samples on the slide is shown in Figure 2. The boundaries for the individual clusters are set as ± 3 SD from the mean AF within each cluster. This control was used to increase stringency and thus data points that fall outside clusters are classified as "no call".

To investigate the variability between tag sequences, each allele-specific extension primer was designed with two alternative tag sequences. The cluster diagrams for each of the primer pair combinations were compared (data not shown) and all combinations gave similar clusters as compared to the diagrams presented in Figure 2, indicating that the cluster distributions were mainly related to the extension rather than the hybridization properties of the tag sequence. However, for ITGB3 the clusters were shifted towards the left but functional when using one of the primer pairs. This can be due to either differences in hybridization efficiency or failure in the primer synthesis.

In addition, in order to investigate the effect of protease on genotyping calls, eight samples were genotyped in the presence and absence of protease. Without protease (ASE), correct clustering could be obtained for 8 out of the 10 SNPs whereas with protease (PrASE) correct clustering was obtained for all SNPs. The SNPs that did not render 3 distinguishable clusters by ASE are located in the ITGB3 and FGB genes (Figure S1 from Additional File 1). In these cases, the mismatch primer was mistakenly extended for one of the homozygous types, making these samples appear as heterozygotes. The Pyrosequencing assay was employed on these SNPs, confirming the PrASE results. In addition, in the remaining 8 SNPs, the inclusion of protease renders complete partitioning of the clusters by increasing the distance between clusters, indicating the higher robustness of PrASE. These findings are consistent with previous reports indicating lack of specificity of the ASE assay [9,13,21,22].

Genotyping of 442 samples (4420 genotypes) was performed side-by-side with PrASE and Pyrosequencing to investigate the accuracy of the methods. All loci were amplified in an outer 10-plex PCR followed by an inner 10-plex PCR for genotyping with PrASE and also 10 inner simplex PCR reactions for genotyping with Pyrosequencing. Pyrosequencing was performed as previously described [17]. A 99.8 % concordance was achieved between the two assays. Eight discordant genotypes were observed and these results were evenly distributed among all SNPs and PCR-plates (Table 2). Five of the ambiguities were settled with Sanger DNA sequencing as a third independent method, unfortunately there were no remaining genomic DNA of the last three samples. PrASE was correct in four of the cases and Pyrosequencing in one case.

**Figure 3:**
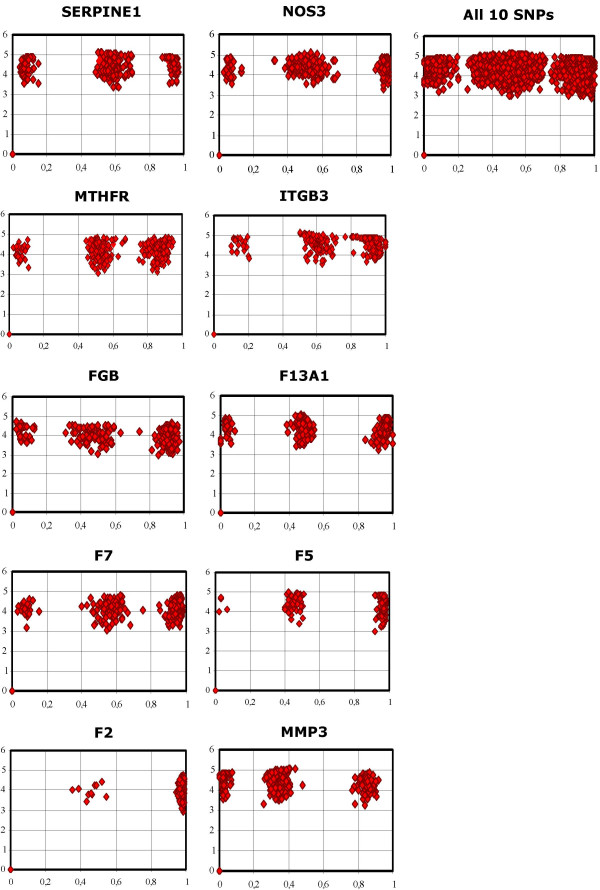
**Robustness of the assay**. All the 442 typed samples from all experiments plotted together for each of the SNPs. As illustrated, clusters of each SNP give the same pattern between runs indicating the robustness and consistency of the assay. In fact, the entire dataset can be clustered in one diagram (right panel), still forming three distinct and correct clusters.

The robustness of the PrASE technology could be demonstrated by examining the genotyping data for all 442 samples in the same cluster diagrams (See Figure 3). Each SNP gave a distinct individual pattern that is very reproducible between runs (48 samples at each run). No traces of significant contamination is visible, in such a case the clusters would be indistinguishable. In fact, as seen in early results, before good conditions for the silicone rubber mask that divides the slide into 48 wells was found, a contamination in a sample is obvious in its placement between clusters for several SNPs. This is a clear advantage of a multiplex detection system as opposed to the singleplex of Pyrosequencing where a contamination is not always as obvious. Furthermore, as indicators of contaminations five negative and one positive controls were included in each 96-well PCR plate. The negative controls typically gave lower signal intensities than positives and positioned themselves between clusters.

**Figure 4:**
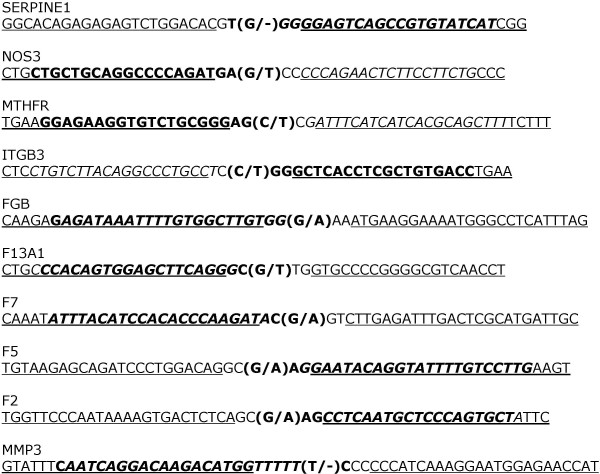
**Sequences of all PCR amplicons used for PrASE**. Inner multiplex PCR primers are underlined. The placement of PrASE and Pyrosequencing primers are indicated in bold and italic respectively. Note that the forward strand sequences are displayed hence the actual sequences of primers are reverse and complementary where appropriate.

In fact, all 10 SNPs can be combined into a single plot of 4420 genotypes (Figure 3 right panel) and still form three distinct clusters. Nevertheless, the obvious differences in cluster patterns between different SNPs can be explained by variations in sequence context (Figure 4), affecting hybridization of extension primers to the target molecules and to the spotted signature tags. The variation in MMP3 is a 5T/6T insertion/deletion that may be difficult to analyze due to 3'-terminus instability of hybridized probes to this locus. In fact, MMP3 works very well considering that one of the allele-specific primers ends with six deoxythymidines and the other one ends with five deoxythymidines and a deoxycytidine theoretically giving the primers very different duplex stabilities at the 3'-terminus.

In addition to accuracy and robustness, the reproducibility of the method was investigated by analyzing 24 samples. The investigated samples were all derived from the same PCR reactions and divided into two PrASE reactions followed by hybridization to one microarray slide. Standard deviations (SDs) were calculated between the two allelic fractions for each sample. The mean SD was 0.018 for all SNPs while for the individual SNPs, the mean SD ranged between 0.0047 and 0.030. Furthermore, 12 samples were assayed twice on separate dates (four months apart and with different inner PCR reactions, batches of microarray slides, enzymes and reagents). A mean SD of 0.023 was obtained for the two separate runs and for the individual SNPs the SD ranged between 0.0054 and 0.039. The results here show that there is very little inter and intra chip variability proving the reproducibility of the assay. In addition, low SDs reflects tightly held clusters (see Figure 1).

## Conclusion

As a complement to whole-genome SNP typing technologies, where a large number of SNPs are examined in each sample, there is an important niche for technologies that accurately can type a large number of samples in not as many SNPs. In this work, genotyping of ten polymorphisms associated with thrombosis formation was performed with PrASE and 99.8% concordance was met when data was compared to Pyrosequencing. However, the PrASE assay proved to be considerably less labor intensive due to its multiplexing capability in both PCR amplification and genotyping. Yet, the number of investigated SNPs per sample may be further increased by design and addition of more signature tags on the arrays.

There is a plentitude of genotyping technologies with similar multiplexing and sample capabilities as PrASE. Some have been commercialized and are available in with specialized instruments and kits which naturally reduce the complexity for the user but at the same time increases costs and reduces the degrees of freedom for the researcher. Some such as PrASE have only been described academically and it is therefore difficult to get a simple price quote but in this particular case running costs is in the range of 0.15 USD per SNP.

Some other techniques in the same applicaton niche as PrASE are limited in multiplexing capacity by the technique itself, such as Pyrosequencing and various real time PCR assays (5' nuclease assay or TaqMan [23] and molecular beacons [24]), whereas others are limited by the amplification method, such as single-base extension (SBE) [10] with microarray [25] or MALDI-TOF MS [26] detection and PrASE. With MS detection, SBE has been limited to 30-plex detection due to a limited number of mass tags available or the resolution of the system [27]. The similar microarray platforms used for SBE and PrASE would most likely be of similar multiplexing levels except that PrASE uses the double amount of primers (a negligible cost in the case for many samples and moderate number of SNPs) and thus uses double the amount of spots on the microarray whereas SBE instead uses a two or four color detection hence a more expensive scanner. The multiplexing level for PrASE or conventional allele-specific extension (ASE) and SBE seems to be much larger than previously anticipated; the same researchers have compared 650 SNPs with ASE and SBE [28] and both methods are scalable to hundreds of thousands of SNPs in a single reaction [29]. The premises upon which these were chosen are not clear and it is our belief that PrASE technology can offer significant improvements in terms of accuracy and robustness and thereof increase the number of typeable SNPs, *i.e*. a more flexible choice in SNPs. This is especially important since the most common biallelic variations in the human genome is the C-T and the G-A transitions that are also the most difficult polymorphisms to type by allele specific extensions if not the PrASE technology is employed.

## Methods

### SNPs

Ten SNPs and single base insertions/deletions in as many genes were selected that have been suggested as prothrombotic genetic variations. Gene names, abbreviations and GenBank accession numbers as well as polymorphism positions and types can be found in Table 1. Note that the polymorphisms in SERPINE1 and MMP3 are single base insertions/deletions. The SERPINE1 variation is a 4 or 5 deoxyguanosine residues while the MMP3 variation is a 5 or 6 deoxythymidine residues.

### Patients

DNA was extracted from blood from unrelated individuals of Caucasian/Scandinavian origin (from a cohort of patients presenting with symptoms of acute chest pain) [17]. The patients were included in the Carlscrona Heart Attack Prognosis Study approved by the ethics committee at the University of Lund, Sweden in compliance with the Declaration of Helsinki. Each 96-well PCR plate also contained five negative water controls and one positive control (Clontech Laboratories, Palo Alto, CA, USA). To prevent contamination problems three semi-clean rooms with limitations to the DNA allowed in the rooms were used.

### Outer PCR

A nested multiplex amplification of the genomic regions was performed. The same outer PCR was used as template both for 10 separate inner PCRs for Pyrosequencing as well as an inner multiplex PCR, used for PrASE. All primers for PCR were designed from GenBank entries and searched for specificity and were synthesized by MWG-Biotech (Ebersberg, Germany) (Table S1 from Additional File 1). The outer PCR was optimized by running gradient PCRs and simplex inner PCRs. An equivalent of 1–5 ng genomic DNA was used for each 25 μl reaction with 0.1 μM of each primer (except for the MTHFR-, F5- and F2-regions which needed 0.14 μM). The PCR contained 2 mM MgCl_2_, 0.2 mM dNTP (Amersham Biosciences, Uppsala, Sweden) and 0.5 U AmpliTaq Gold with 1× PCR Gold buffer (Applied Biosystems, Foster City, CA). The amplification program was 94°C for 12 min followed by 35 cycles at 94°C 50 s, 65°C 30 s and 72°C 2 min and finally 72°C for 10 min and it was performed on a GeneAMP thermocycler (PE Biosystems, Foster City, CA).

### Inner Simplex PCRs for Pyrosequencing

0.5 μl of the outer PCR was used as template to separately amplify each SNP region in inner PCRs with the same concentrations as above but using 0.2 U polymerase. One primer in each pair was biotinylated for later immobilization. Amplification program were as above with the exceptions of 30 s of denaturation in each cycle and annealing temperatures of 64.5°C for all SNPs but FGB which annealed at 60°C and it was performed on a MWG multi block thermocyclers (MWG-Biotech).

### Inner Multiplex PCR for PrASE

0.5 μl of the outer PCR was used as template to amplify all 10 loci in 50 μl inner PCR reaction with the same concentrations as above except 0.04 μM of each of the 20 primers and using 1 U of Platinum Taq DNA polymerase with 1× PCR buffer (Invitrogen AB, Lidingö, Sweden). Primers are indicated in Table S1 from Additional File 1 and one primer in each pair was biotinylated for immobilization. The amplification program was 94°C for 5 min followed by 45 cycles at 94°C 30 s, 60°C 30 s and 72°C 30 s and finally 72°C for 10 min and it was performed on a GeneAMP thermocycler (PE Biosystems).

### Pyrosequencing

Single stranded DNA was generated by the use of immobilization of the biotinylated PCR products to 50 μg of streptavidin coated super paramagnetic beads (Dynabeads M-270, Dynal Biotech, Oslo, Norway) and 1.65 pmol Pyrosequencing primer (Table S1 from Additional File 1) was hybridized by the use of a Magnatrix 1200 pipetting robot (Magnetic Biosolutions, Stockholm, Sweden) according to the manufacturers' instructions. Pyrosequencing was performed according to manufacturer's instructions on a PSQ™ 96 HS instrument (Biotage, Uppsala, Sweden) and analyzed with the accompanying SNP software.

### PrASE Reaction

The PrASE assay was automated by the use of a Magnatrix 1200 pipetting robot (Magnetic Biosolutions) that handles magnetic beads used for streptavidin immobilization of the biotinylated PCR products. The robot is capable of handling 48 samples in parallel, which is the same number as can be hybridized to one microarray slide. 200 μg streptavidin-coated super paramagnetic beads (Dynabeads M-280, Dynal Biotech) were used for each inner multiplex PCR product. Immobilization and washes between steps were made according to the manufacturer's instructions and as described before [14]. Single-stranded DNA was prepared by alkali treatment and annealed to allele-specific extension primers (0.08 μM in 60 μl) (Table S2 from Additional File 1). The PrASE reaction was performed at 37°C in a total volume of 60 μl. containing 1× extension buffer (42.5 mM Tris-HCl pH 8, 5 mM MgCl_2 _and 1 mM DTT), 0.25 % bovine serum albumin and 10 U DNA polymerase (3'-5' exonuclease deficient Klenow fragment, Fermentas, Helsingborg, Sweden). The PrASE reaction was started by simultaneous addition of 1.5 μM of each dNTP (Amersham Biosciences) and 20 μg Proteinase K (Invitrogen). 50 % of the dCTP and dUTP were Cy5 labeled to allow fluorescence detection of extended primers. Strand-specific alkali elution of the primers was made before hybridization to the tag-microarray.

### Tag Microarrays

Tag microarrays were prepared as previously reported [30]. Forty-eight oligonucleotides (MWG-Biotech) were spotted (Q-array, Genetix, Hampshire, United Kingdom) in triplicates onto glass slides (Code Link, Amersham Biosceinces, Uppsala, Sweden). The oligonucleotide pattern was repeated on each slide and these sub-arrays were separated during hybridization using a silicone mask to facilitate parallel analysis of 48 samples [31]. Hybridization of the extended allele-specific primers was performed at 50°C for 1 h. Each primer contained a specific tag at its 5'-end complementary to one of the 48 spotted oligonucleotides. The slides were washed according to the manufacturer before scanning (Agilent scanner, Agilent Technologies, Palo Alto, CA, USA). Data was extracted with GenePix 5.0 software (Axon instruments, USA) and analyzed with a custom Microsoft Excel script.

### Sanger DNA sequencing

Conflicting results were resolved using Sanger dideoxy sequencing with BigDye terminator chemistry (Applied Biosystems, Foster City, CA) and an ABI 3700 Analyzer instrument (Applied Biosystems). The same PCR setups as for Pyrosequencing were used and the inner PCR primers were used as sequencing primers.

## Authors' contributions

MK participated in design of the study, carried out PCR and PrASE reactions, analysis of the results and drafted the manuscript. EH participated in design of the study and carried out PCR and PrASE reactions. KH, MLP and JO carried out PCR reactions and Pyrosequencing. JL and AA conceived of the study and helped to draft the manuscript. All authors read and approved the final manuscript.

## Supplementary Material

Additional File 1 Contains figure S1 with legend Tables S1 and S2. Click here for file

## Figures and Tables

**Table 1:** Genotyped polymorphisms

**Gene (alternative name)**	**Abbreviation**	**DbSNP number**	**Polymorphism position (nt)**	**PrASE amplicon lenght**	**PrASE amplicon GC content**
serpin peptidase inhibitor, clade E (plasminogen activator inhibitor type 1), member 1	SERPINE1	rs1799768	-675 (4G/5G)	48/49	0.61
nitric oxide synthase 3	NOS3	rs1799983	894 (G/T)	48	0.64
5,10-methylenetetrahydrofolate reductase	MTHFR	rs1801133	667 (C/T)	53	0.48
integrin, beta 3 (platelet glycoprotein IIIa)	ITGB3	rs5918	196 (T/C)	49	0.62
fibrinogen beta chain	FGB	rs1800791	-854 (G/A)	56	0.38
coagulation factor XIII, A1 polypeptide	F13A1	rs5985	163 (G/T)	46	0.69
coagulation factor VII	F7	rs510317	-402 (G/A)	56	0.40
coagulation factor V	F5	rs6025	1691 (G/A)	54	0.45
coagulation factor II (thrombin)	F2	rs1799963	20210 (G/A)	53	0.48
matrix metallopeptidase 3 (stromelysin 1)	MMP3	rs3025058	-1675 (5T/6T)	57/58	0.42

**Table 2:** Conflicting results

**SNP**	**PCR Plate**	**Well**	**PrASE**	**Pyrosequencing**	**Sanger DNA sequencing**
NOS3	A15	A5	AC	AA	nr
NOS3	A15	A8	CC	AC	nr
F7	A19	F9	AG	AA	AG
FGB	A19	H9	GG	AG	AG
F7	A21	G10	AG	AA	AG
SERPINE1	A21	H7	AA	CA	nr
FGB	A24	B6	GG	AG	GG
FGB	A24	C6	GG	AG	GG

Note: nr = not resolved
