# Supplementary material for: Comparison of PrASE and Pyrosequencing for SNP Genotyping
Source: BMC Genomics. 2006 Nov 16;7:291. doi: 10.1186/1471-2164-7-291 (PMC1657021; doi:10.1186/1471-2164-7-291)
Supplement: Additional File 1 — Contains figure S1 with legend Tables S1 and S2. [file 1471-2164-7-291-S1.doc]

# Additional File 1

###

### Figure S1. Comparison of results with and without inclusion of protease

Two SNPs (ITGB3 and FGB) gave false positive signals for one of the allele-specific extension primers when protease was not included in the extension reaction (ASE). Hence, one of the homozygous genotypes wrongly clustered as heterozygous. When protease was included these two genotypes were correctly separated (PrASE).

### Table S1. Primers used

| **Name** | **Sequence** |
| --- | --- |
| SERPINE1_iPCR_FoB | bio-GGCACAGAGAGAGTCTGGACAC |
| SERPINE1_iPCR_Re | CCGATGATACACGGCTGACTCC |
| NOS3_iPCR_Fo | CTGCTGCTGCAGGCCCCAGAT |
| NOS3_iPCR_ReB | bio-GGGCAGAAGGAAGAGTTCTGGG |
| MTHFR_iPCR_Fo | TGAAGGAGAAGGTGTCTGCGGG |
| MTHFR_iPCR_ReB | bio-AAAGAAAAGCTGCGTGATGATGAAAT |
| ITGB3_iPCR_FoB | bio-CTCCTGTCTTACAGGCCCTGCC |
| ITGB3_iPCR_Re | TTCAGGTCACAGCGAGGTGAGC |
| FGB_iPCR_Fo | CAAGAGAGATAAATTTTGTGGCTTGT |
| FGB_iPCR_ReB | bio-CTAAATGAGGCCCATTTTCCTTCAT |
| F13_iPCR_Fo | CTGCCCACAGTGGAGCTTCAGG |
| F13_iPCR_ReB | bio-AGGTTCACGCCCCGGGGCAC |
| F7_iPCR_Fo | CAAATATTTACATCCACACCCAAGAT |
| F7_iPCR_ReB | bio-GCAATCATGCGAGTCAAATCTCAAG |
| F5_iPCR_FoB | bio-TGTAAGAGCAGATCCCTGGACAG |
| F5_iPCR_Re | ACTTCAAGGACAAAATACCTGTATTC |
| F2_iPCR_FoB | bio-TGGTTCCCAATAAAAGTGACTCTCA |
| F2_iPCR_Re | GAATAGCACTGGGAGCATTGAGG |
| MMP3_iPCR_Fo | GTATTTCAATCAGGACAAGACATGG |
| MMP3_iPCR_ReB | bio-ATGGTTCTCCATTCCTTTGATGGG |
| HOKI9 | CAAGTGATTCTCCTGCCTCAACCT |
| HOKI10 | CTCCACTGTTTCTTCCTGGAATTC |
| HOKI7 | CTCTGTTCTCCTTGTCCTCATATC |
| HOKI8B | bio-GGCACCTGGCCTAAAGACATTTTA |
| HOKI6T | AATCAGGACAAGACATGGTTTTTT |

### Notes: bio indicates biotinylations. HOKI9 and 10 are for outer PCR, HOKI7 and 8B are for inner PCR for Pyrosequencing and HOKI6T is for Pyrosequencing.

### Table S2. Extension primers

| **Name** | **Sequence** |
| --- | --- |
| L4_SERPINE1_C | TTAGTCTCCGACGGCAGGCTTCAATATGATACACGGCTGACTCCCCC |
| L5_SERPINE1_A | CTGTGACAGAGCCAACACGCAGTCTATGATACACGGCTGACTCCCCA |
| L6_NOS3_G | CCTGGTGGTTGACTGATCACCATAACTGCTGCAGGCCCCAGATGAG |
| L7_NOS3_T | GCATGTATAGAACATAAGGTGTCTCCTGCTGCAGGCCCCAGATGAT |
| L8_MTHFR_C | GCTAGATGAAGAGCAAGCGCATGGAGGAGAAGGTGTCTGCGGGAGC |
| L9_MTHFR_T | TACAACCGACAGATGTATGTAAGGCGGAGAAGGTGTCTGCGGGAGT |
| L10_ITGB3_A | TTCAATCTGGTCTGACCTCCTTGTGGGTCACAGCGAGGTGAGCCCA |
| L11_ITGB3_G | ACACGATGTGAATATTATCTGTGGCGGTCACAGCGAGGTGAGCCCG |
| L14_FGB_G | GGCAACTCATGCAATTATTGTGAGCAGAGATAAATTTTGTGGCTTGTGGG |
| L15_FGB_A | CCAGAAGTATATTAATGAGCAGTGCAGAGAGATAAATTTTGTGGCTTGTGGA |
| L16_F13A1_G | AAGCAGTCTGTCAGTCAGTGCGTGAACCACAGTGGAGCTTCAGGGCG |
| L17_F13A1_T | AATGATGCTCTGCGTGATGATGTTGCCACAGTGGAGCTTCAGGGCT |
| L18_F7_G | AATACACGAAGGAGTTAGCTGATGCATTTACATCCACACCCAAGATACG |
| L19_F7_A | GCTGTTAATCATTACCGTGATAACGCCATTTACATCCACACCCAAGATACA |
| L22_F5_C | TTACCTATGATTGATCGTGGTGATATCCGCAAGGACAAAATACCTGTATTCCTC |
| L23_F5_T | GCTGTGGCATTGCAGCAGATTAAGGCAAGGACAAAATACCTGTATTCCTT |
| L24_F2_C | TGACGTCATTGTAGGCGGAGAGCTAAGCACTGGGAGCATTGAGGCTC |
| L25_F2_T | TCAATAATCAACGTAAGGCGTTCCTAGCACTGGGAGCATTGAGGCTT |
| L26_MMP3_T | TTATCGGCTACATCGGTACTGACTCCAATCAGGACAAGACATGGTTTTTT |
| L27_MMP3_C | CCATTATCGCCTGGTTCATTCGAGTCAATCAGGACAAGACATGGTTTTTC |
| L28_SERPINE1_C | GGCGTACCTTCGCGGCAGATATAATATGATACACGGCTGACTCCCCC |
| L29_SERPINE1_A | AACTGAGCCGTAGCCACTGTCTGTCCATGATACACGGCTGACTCCCCA |
| L30_NOS3_G | GTTGTGCTGAATTAAGCGAATACCGCTGCTGCAGGCCCCAGATGAG |
| L31_NOS3_T | TTATATCTGCACAACAGGTAAGAGCCTGCTGCAGGCCCCAGATGAT |
| L32_MTHFR_C | AAGAGGCGGCGCTTACTACCGATTCGGAGAAGGTGTCTGCGGGAGC |
| L33_MTHFR_T | CGGTCACACGTTAGCAGCATGATTGGGAGAAGGTGTCTGCGGGAGT |
| L34_ITGB3_A | AAGAAGAGTCAATCGCAGACAACATGGTCACAGCGAGGTGAGCCCA |
| L35_ITGB3_G | CATATCGCGCTGTGACGATGCTAATGGTCACAGCGAGGTGAGCCCG |
| L38_FGB_G | AACCTAACATTGATTCAGGTACAGGAGAGATAAATTTTGTGGCTTGTGGG |
| L39_FGB_A | CAGTTGATCATCAGCAGGTAATCTGGAGAGATAAATTTTGTGGCTTGTGGA |
| L40_F13A1_G | ATATGTTATCTGCCACGCCGATTATCCACAGTGGAGCTTCAGGGCG |
| L41_F13A1_T | AACTGGATACGATTGGATTCGACAACCACAGTGGAGCTTCAGGGCT |
| L42_F7_G | CATCGTCAACGACGTTCTCATGGTTATTTACATCCACACCCAAGATACG |
| L43_F7_A | CCATTCCAGACATGCTCGTTGAAGCATTTACATCCACACCCAAGATACA |
| L44_MMP3_T | GCCATCGCTGGACTATCGAAGAGTGCAATCAGGACAAGACATGGTTTTTT |
| L45_MMP3_C | ATCTCGTTCCGTATCGCGTCGAACTCAATCAGGACAAGACATGGTTTTTC |
| L46_F5_C | GAACGCAATATTCACAAGCAATGCGCAAGGACAAAATACCTGTATTCCTC |
| L47_F5_T | AAGAGACCGCGACTTACCATGTATCCAAGGACAAAATACCTGTATTCCTT |
| L48_F2_C | GCAGAACTGATGAGCGATCCGAATAAGCACTGGGAGCATTGAGGCTC |
| L49_F2_T | AACCTTCAACTACACGGCTCACCTGAGCACTGGGAGCATTGAGGCTT |
